# Novel likely pathogenic variant in *NR5A1* gene in a Tanzanian child with 46,XY differences of sex development, inherited from the mosaic father

**DOI:** 10.1530/EDM-22-0384

**Published:** 2023-04-26

**Authors:** Rahim Karim Damji, Mohamed Zahir Alimohamed, Hedi L Claahsen-van der Grinten, Dineke Westra, Ben Hamel

**Affiliations:** 1 Department of Paediatrics, Regency Medical Centre, Dar es Salaam, Tanzania; 2 Shree Hindu Mandal Hospital, Dar es Salaam, Tanzania; 3 Department of Biochemistry, Muhimbili University of Health and Allied Sciences, Dar es Salaam, Tanzania; 4 Department of Genetics, University Medical Center Groningen, Groningen, The Netherlands; 5 Tanzania Human Genetics Organization, Dar es Salaam, Tanzania; 6 Department of Paediatric Endocrinology, Amalia Children’s Hospital, Radboud University Medical Center, Nijmegen, The Netherlands; 7 Department of Human Genetics, Radboud University Medical Center, Nijmegen, The Netherlands

**Keywords:** Paediatric, Male, Black - African, Tanzania, United Republic of, Adrenal, Adrenal, Unique/unexpected symptoms or presentations of a disease, April, 2023

## Abstract

**Summary:**

Pathogenic variants in the nuclear receptor subfamily 5 group A member 1 gene (*NR5A1*), which encodes steroidogenic factor 1 (SF1), result in 46,XY and 46,XX differences of sex development (DSD). In 46,XY individuals with a pathogenic variant in the *NR5A1* gene a variable phenotype ranging from mild to severe is seen, including adrenal failure, testis dysgenesis, androgen synthesis defects, hypospadias and anorchia with microphallus and infertility. We report the clinical, endocrinological and genetic characteristics of a patient with 46,XY DSD with a novel likely pathogenic missense variant in the *NR5A1* gene. A retrospective evaluation of the medical history, physical examination, limited endocrinological laboratory analysis and genetic analysis with DSD gene panel testing was performed. A 1.5-month-old individual was referred with ambiguous genitalia. The karyotype was 46,XY. The endocrinological analyses were within normal male reference including a normal response of cortisol within an adrenocorticotropic hormone test. A novel heterozygous missense variant c.206G>C p.(Arg69Pro) in the *NR5A1* gene was detected. This variant was present in mosaic form (~20%) in his unaffected father. Because another missense variant at the same position and other missense variants involving the same highly conserved codon have been reported, we consider this *NR5A1* variant in this 46,XY DSD patient as likely pathogenic in accordance with the ACMG/AMP 2015 guidelines causing ambiguous genitalia but no adrenal insufficiency. This variant was inherited from the apparently unaffected mosaic father, which might have implications for the recurrence risk in this family.

**Learning points:**

- The importance of performing trio (patient and parents) sequencing is crucial in pointing out the origin of inheritance.
- In a 46,XY differences of sex development patient, a normal adrenal function does not rule out an *NR5A1* mutation.
- With the support of a dedicated overseas institute partnership, we could solve this complex clinical case by molecular diagnosis in a resource-limited setting.

## Background

Differences of sex development (DSD) have been defined as ‘congenital conditions in which the development of chromosomal, gonadal, or anatomical sex is atypical’. Therefore, the term DSD constitutes a spectrum of disorders that affect the genitourinary tract and the endocrine-reproductive system. Based on chromosome karyotype, DSD are usually classified into 46,XX DSD, 46,XY DSD and sex chromosome DSD (1).

Heterozygous pathogenic *NR5A1* variants account for 10–20% of 46,XY DSD cases (2). *NR5A1* gene encodes for Steroidogenic factor 1 (SF1), which functions as a transcription factor for sex determination as well as regression of Mullerian structures (3).

Since the description of the first 46,XY DSD patient with adrenal insufficiency and a pathogenic *NR5A1* variant (4), the spectrum of phenotypes associated with pathogenic *NR5A1* variants has greatly expanded. It became clear that adrenal insufficiency is a fairly rare feature of 46,XY DSD (2). Up to 2019, more than 180 pathogenic *NR5A1* variants have been reported (3).

There is no clear phenotype–genotype correlation and there is wide phenotypic variability between and within families (2). The spectrum of genital anomalies in 46,XY DSD patients carrying *NR5A1* variants comprises partial to complete gonadal dysgenesis with female external genitalia, genital ambiguity, penoscrotal hypospadias, micropenis, cryptorchidism, anorchia and male factor infertility (5). Heterozygous pathogenic *NR5A1* variants can also cause different types of ovarian insufficiency in 46,XX individuals (6).

Here, we describe clinical and molecular findings in an individual with 46,XY DSD born with ambiguous genitalia and with a normal adrenocortical function and in whom a novel heterozygous *NR5A1* variant was identified, inherited from the apparently unaffected mosaic father.

## Case presentation

**Figure 1:**
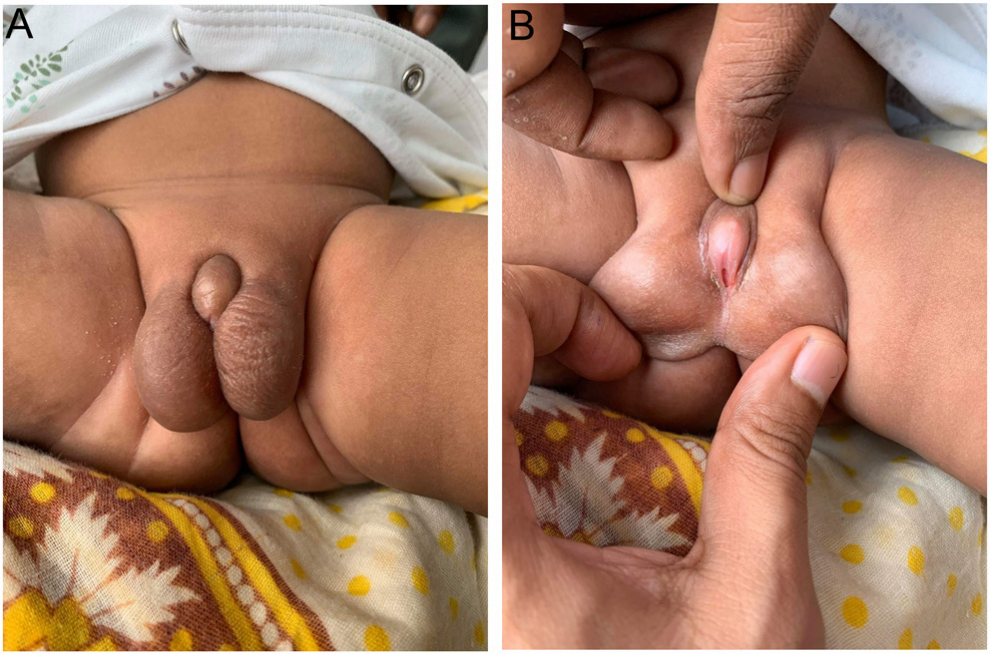
(A) Bifid scrotum, small phallus. (B) Curved phallus, single perineal opening, proximal hypospadias and visible/palpable gonads.

The infant was 1.5 months old when he first presented at our centre with ambiguous genitalia. He was born at term to nonconsanguineous parents, after an uneventful pregnancy with a birth weight of 3000 g. He was the only child in the family. As far as parents could tell, there were no cases of ambiguous genitalia and sub- or infertility in their families. Father was not known for hypospadias and from history, no complaints of adrenal insufficiency. Physical examination of the child revealed a small phallus (stretched length 2 cm), proximal hypospadias with the curvature of the phallus and single perineal orifice, bifid scrotum and bilateral palpable gonads in the scrotal sac (1 mL bilaterally) (Fig. 1A and B). No extra-genital anomaly was detected. Otherwise, the physical examination was unremarkable.

## Investigation

Karyotyping revealed a normal 46,XY karyotype and male gender was assigned. Mullerian derivatives could not be visualized on pelvic ultrasonography. Adrenal insufficiency was excluded with a standard dose adrenocorticotropic hormone (ACTH) stimulation test, which revealed a normal peak cortisol level. Other laboratory results were all within the reference levels of age for 46,XY individuals. The testosterone level at the age of 6 weeks (mini puberty) is expected to be higher in normal infants; however, testosterone here is low as expected in a child with gonadal dysgenesis and insufficient testosterone leading to incomplete virilization (Table 1).

**Table 1:** Laboratory tests and their results at the age of 6 weeks.

Laboratory tests	Results	Reference values*
Follicle stimulating hormone, IU/L	4.6	0.1–11.3
Luteinising hormone, IU/L	0.1	0.02–8.0
Testosterone, nmol/L	0.1	0.03–6.14
Dihydrotestosterone, ng/mL	0.18	0.06–0.30
17-OH progesterone, ng/mL	1.3	0.4–2.0
Androstenedione (LC-MS), ng/mL	1	0.6–2.0
Sodium, mmol/L	135	135–145
Potassium, mmol/L	4.4	4.1–5.3
Random blood glucose, mmol/L	5.1	3.3–7.8
Peak cortisol level, µg/dL	23.3	>18

*Male reference values.

After informed consent was obtained, blood samples were collected from the patient and his parents and shipped to Genome Diagnostics of the Department of Human Genetics, Radboud University Medical Center, Nijmegen, The Netherlands, for trio exome sequencing, as described previously (7). In short, exome enrichment was performed with the Agilent SureSelectQXT Human All Exon v5 Kit. Read alignment was done with BWA, and variant calling with GATK (SNVs) and CoNIFER (CNVs). After that, variants were annotated using an in-house developed pipeline. A bioinformatic filter for our ‘DSD’ gene panel (version DG-3.0) was applied and variants were selected and prioritized (information about the prioritization is available on request). Panel content and gene coverage can be found on the website of the Department of Human Genetics of the Radboud University Medical Center (https://order.radboudumc.nl/en/products/wes-disorders-differences-of-sex-development-dsd-primary-adrenal-insufficiency).

A heterozygous variant in the *NR5A1* gene was found in the patient:

*NR5A1* Chr9(GRCh37):g.127265396C>G NM_004959.5:c.206G>C p.(Arg69Pro).

This exact *NR5A1* variant has not been reported before and is not present in control populations of the Genome Aggregation Database (www.gnomad.org; v.2.1.1), but another missense change at the same nucleotide 206 and one at the same highly conserved amino acid arginine at position 69 in *NR5A1* has been published in multiple patients (Table 2; 8, 9, 10) and functional analysis of these variants showed decreased protein expression (8). Therefore, we consider this variant as likely pathogenic according to the ACMG/AMP 2015 guidelines (11) (Table 3). With exome sequencing, the same variant was found in the father in mosaic form (~17%), which was confirmed with Sanger sequencing (present in ~20% of his DNA), whilst in the mother this variant was not identified. No other tissue was tested in the father to determine the mosaic status in there.

**Table 2:** Previously reported *NR5A1* variants involving amino acid 69.

Reference	Patient	Nucleotide change	Amino acid change	Variant class	Clinical data	Family
This study	Present case	c.206G>C	p.Arg69Pro	Likely pathogenic	Bifid scrotum, small penis, curved phallus, single perineal opening, proximal hypospadias	From mosaic father not exhibiting this phenotype
Na *et al.* (8)	Patient 6	c.206G>A	p.Arg69His	Pathogenic	Bilateral dysplastic inguinal testes	From father with likely normal phenotype
Na *et al.* (8)	Patient 5	c.205C>G	p.Arg69Gly	Pathogenic	Bifid poorly developed scrotum, small penis, inguinal testes	From mother
Costanzo *et al.* (9)	Patient from Family 5	c.206G>A	p.Arg69His	Pathogenic	No data	Familial case, so either from mother or father
Kim *et al.* (10)	Patient 15	c.205C>G	p.Arg69Gly	Pathogenic	Clitoromegaly, atrophic testes in pelvic cavity	Sporadic and no segregation analysis was performed

**Table 3:** Applied ACMG/AMP 2015 criteria for the detected *NR5A1* variant.

Rule	Description	Evidence/justification to support the use of rule
PM1	Located in a mutational hotspot and/or critical and well-established functional domain without benign variation	Located in the Zinc finger, nuclear hormone receptor-type protein domain of Steroidogenic factor 1, in which many pathogenic variants have been described; no non-synonymous variants present in control populations in the Genome Aggregation Database (gnomAD v2.1.1) between amino acids 12 and 82.
PM2	Absent from controls (or at low frequency if recessive)	Absent from all control populations in the Genome Aggregation Database (gnomAD v2.1.1))
PM5	Novel missense at an amino acid residue where a different amino acid has been determined to be pathogenic has been seen before	- c.206G>A p.(Arg69His) described in (8) and (9)
		- c.205C>G p.(Arg69Gly) described in (8) and (10)
PP3	Multiple lines of computational evidence support a deleterious effect on the gene or gene product	Align GVGD (v2007): Class C65
		PolyPhen2: HDivPred: probably damaging (score: 1).	HVarPred: probably damaging (score: 1)	SIFT (v6.2.0): DELETERIOUS (score: 0.00, median: 2.98)	MutationTaster (v2021): Deleterious.

## Treatment

No pharmacological treatment was offered to this child since all the biochemical investigations were within normal range. Surgical correction of his external genitalia was advised.

## Outcome and follow-up

Periodic follow-up was advised to monitor any new endocrine-related problems. The patient remained asymptomatic 12 months post-diagnosis.

## Discussion

We describe a 46,XY DSD patient born with ambiguous genitalia with a novel heterozygous variant in the *NR5A1* gene, which was inherited from the mosaic unaffected father. Pathogenic variants in *NR5A1* are associated with 46,XY sex reversal 3 (OMIM 612965). In the majority of cases, these are heterozygous variants. The *NR5A1* variant in our patient has not been reported before, but another missense change at the same nucleotide position 206 has been published in multiple patients (Table 2). More variants involving changes in the highly conserved amino acid arginine at position 69 in 46,XY DSD patients are described in the literature (Table 2). This variant is located in a functional domain, not present in the control population of the Genome Aggregation Database (www.gnomad.org; 12) and multiple lines of computational evidence support a deleterious effect on the gene product. Therefore, we consider this variant as likely pathogenic according to the ACMG/AMP 2015 guideline (11).

As adrenal insufficiency is described in some but not all patients with *NR5A1* mutations, adrenal testing is recommended. Our patient had a normal response to ACTH excluding adrenal insufficiency.

Mosaicism of *NR5A1* variants has been described before in two non-affected fathers (13, 14). Both fathers had normal genitalia and the mosaicism was confirmed by using another sequencing technique (13) or confirmed in another tissue (14). Identification of mosaicism in a parent has potential consequences for genetic counselling and the recurrence risk when this variant is also present in the germ cells. If present in a high percentage of germ cells, there is a high risk of having other affected sons (46,XY). There might also be a risk of primary ovarian insufficiency (POI) for affected daughters (46,XX). In about 20–30% of 46,XY DSD cases, *NR5A1* variants are inherited from non-affected or later affected mothers (3, 15) and less frequently non-mosaic asymptomatic and sometimes symptomatic (hypospadias) fathers transmit the *NR5A1* variant to their children (3).

Since the first 46,XY DSD patient with a pathological *NR5A1* variant was described by Achermann *et al.* (4), the spectrum of clinical presentation has evolved from the mildest to the most severe form including adrenal insufficiency as a potentially life-threatening complication. These cases, as well as their families, need a multidisciplinary team approach, with a focus on the child’s interest. Major aspects to be discussed with the families include gender assignment, endocrine as well as urological and sexual function. Long-term outcomes, risks of infertility and germ cell tumour also need to be discussed. Genetic counselling has to be part of the management, particularly in this case where the father is a mosaic carrier of the likely pathogenic *NR5A1* variant.

In conclusion, we describe a 46,XY DSD patient with ambiguous genitalia and normal adrenal function with a heterozygous likely pathogenic, novel missense variant, c.206G>C p.(Arg69Pro) in the *NR5A1* gene, which was inherited from a mosaic apparently unaffected father. Long-term clinical and hormonal follow-up in this patient is needed to assess the gonadal and adrenal function.

## Declaration of interest

The author declares that there is no conflict of interest that could be perceived as prejudicing the impartiality of the research reported.

## Funding

This study did not receive any specific grant from any funding agency in the public, commercial or not-for-profit sector.

## Patient consent

Written informed consent was obtained from the parents of the patient for the publication of this case report and images. A copy of the written consent is available for the Editor of this journal.

## Patient’s perspective

The parents expressed how the disease affected the child and the family since there is no similar history in the family. This will further create a family and social stigma since he was initially raised as a female until the karyotype results confirmed the male gender. I am very grateful to both local and international health professionals for making this medical mystery solved genetically. We have accepted the results and will raise him as a male.

## Author contribution statement

R Damji and M Alimohamed developed the project design, described the case report and carried out a literature search and wrote the first draft of the manuscript. D Westra and B Hamel performed and described the molecular analysis and gave critical comments on the manuscript. H Claahsen-van der Grinten gave critical comments on the description of the case report and the interpretation of the data. All authors approved the final manuscript as submitted and agree to be accountable for all aspects of the work.
